# Tocilizumab for the treatment of systemic onset JIA: a single centre experience from India

**DOI:** 10.1186/1546-0096-10-S1-A49

**Published:** 2012-07-13

**Authors:** Sujata Sawhney, Manjari Aggarwal

**Affiliations:** 1 Sir Ganga Ram Hospital, New Delhi, India

## Purpose

Systemic onset JIA (SJIA) is a common subcategory of JIA in India and often difficult to treat. Tocilizumab (Actemra), an IL 6 blocker has been shown to be effective in children with SJIA. The drug is available in India for over a year. We have a high burden of infectious diseases here and biologics are therefore used judiciously. We used the drug in SJIA patients to test the efficacy, tolerability and side effect profile.

## Methods

Prospective study on the use of Tociluzimab in SJIA patients. *Inclusion criteria*: All children with SJIA more than three years of age who had persistent disease activity in spite of adequate NSAID, steroids and DMARDs for at least three months. *Exclusion criteria:* Presence of any active infection specifically tuberculosis.

## Results

*Time period of study*: Children with SJIA were treated with Actemra from March 2009 to December 2010.The data was collected on standardized forms at institution of therapy, and then three monthly. Results up to one year follow up are presented. *Demographic data*: Twelve children were given Actemra, girls 4, boys 8. The age range was from 4-19 years. The median age at disease onset was 8 years (range 1-14 years). The median time from disease onset to commencing Actemra was 6.1 years (range 1.3- 12.4 years). *Infection screening*: One child in this group had a positive PPD and Quantiferon Gold, where the Actemra was withheld till the child was given 6 months of anti-tubercular treatment. All children were tested for HIV, Hepatitis B and C, none were positive.*Background treatment for the patients*: Methotrexate in 7, Leflunamide in one, Methotrexate and Leflunamide combination in 4. Eleven of the twelve were on oral steroids when Actemra was commenced. *Infusion protocol and follow up*: Patients received between one and eighteen doses and were given Actemra for one to eighteen months. The duration of follow up was from one to twenty three months. All patients were pre-medicated with hydrocortisone, ceterizine and paracetamol. No infusion reaction was noted in the total of 86 infusions given. Children over 20kg were given 8mg/kg/dose, those <20 kg were given 12mg/kg/dose. Most were given the infusion monthly.One child, who had had an episode of internuclear opthalmoplegia in the past developed a transient episode of diplopia 2 weeks after the first dose of Actemra, the drug was subsequently discontinued. *Effect of Actemra*: 1. Systemic features: nine children had fever at onset of therapy, two had fever at three months and no child had fever thereafter. 2. Arthritis: The mean joint count prior to Actemra was 5.16, 1.18 at three months and 0.2 at six and twelve months respectively. 3. Serial changes in labs, MD global and CHAQ are tabulated.

**Table 1:** 

Time point	Baseline	Three months	Six months	Nine months	Twelve months
Mean Hb gm%	9.9	10.9	12	12.7	13.2
Mean ESR mm/hr	48	23	14	10	07
Mean platelet /mm^3^	471	319	253	230	215
MD global	32	16	13	12	08
CHAQ	1.2	1.05	0.95	0.625	0.625

Pts on pred	11	09	05	02	02

## Conclusion

With careful screening Tocilizumab can be used safely in areas that have a high burden of infections.The systemic features, haemoglobin and ESR respond rapidly, the arthritis responds later. Some children continue to have active disease inspite of this intervention.

## Disclosure

Sujata Sawhney: None; Manjari Aggarwal: None.

